# Anatomical Variations in Circle of Willis in Patients Undergoing CT Cerebral Angiography in a Tertiary Hospital in Nepal: A Descriptive Cross-sectional Study

**DOI:** 10.31729/jnma.5893

**Published:** 2020-12-31

**Authors:** Prajwal Dhakal, Prakash Kayastha, Sharma Paudel, Sundar Suwal, Mohan Raj Sharma, Ram Kumar Ghimire

**Affiliations:** 1 Department of Radiology and Imaging, HAMS hospital, Kathmandu, Nepal; 2 Department of Radiology and Imaging, Tribhuvan University Teaching Hospital, Kathmandu, Nepal; 3 Department of Neurosurgery, Tribhuvan University Teaching Hospital, Kathmandu, Nepal; 4 Department of Radiology, Nepal Mediciti Hospital, Lalitpur, Nepal

**Keywords:** *Circle of Willis*, *CT angiography*, *variations*

## Abstract

**Introduction:**

Variation in Circle of Willis is a commonly encountered entity in patients undergoing Computed Tomography angiography, identification of which is crucial in management of patients with vascular pathologies. The aim of the study was to find out the anatomical variations in Circle of Willis in patients undergoing Computed Tomography cerebral angiography in a tertiary hospital in Nepal.

**Methods:**

This is a descriptive cross-sectional study involving 95 patients using convenient sampling techniques who were sent to the Department of Radiology and Imaging, Tribhuvan University Teaching hospital, for further evaluation of suspected vascular pathologies in the brain from April 2017 to September 2017. Ethical approval was taken from the Institutional Review Committee of the Institute of Medicine with reference number 326 (6-11-E). CT angiographic images of these patients were evaluated for presence of variations in Circle of Willis, aneurysms and other vascular pathologies. Data were analysed using SPSS.

**Results:**

Among 95 subjects included in the study, the anatomical variations in the arteries of Circle of Willis was seen in 52 (54.7%) patients, hypoplastic posterior communicating artery being the most common variation 33 (34.7%). Aneurysm was seen in 22 (23.2%) of cases.

**Conclusions:**

CT Angiography is commonly performed imaging modality for suspected cases of cerebral aneurysms and various other vascular pathologies. Multidetector computed tomography can effectively detect variations in arteries of Circle of Willis, recognition of which is crucial in operative management of vascular pathologies.

## INTRODUCTION

A classic arterial Circle of Willis (CoW) is bilaterally symmetrical and a complete ring of vessels but variations in this typical configuration is often seen.^1^ Status of the circle is crucial in determining the adequacy of the brain circulation during management of cerebral aneurysms and other vascular diseases.^1^

Digital subtraction angiography (DSA) remains the gold standard for detection of intracranial vascular variations and anomalies, however, Computed Tomography (CT) cerebral angiography high sensitivity and specificity of 81% - 90% and 93%, respectively.^2^ Also computed tomography angiography (CTA) is less invasive and is less time consuming compared to DSA.^3^ Furthermore radiation exposure in imaging the cerebral vessels is significantly lower for CTA (0.67 mSv for CTA compared to 2.71 mSv for DSA).^4^

In this study, we aimed to determine the anatomic variations of CoW seen among the patients undergoing CT cerebral angiography in a tertiary hospital.

## METHODS

This was a descriptive cross-sectional study done in Tribhuvan University Teaching Hospital (TUTH) from April 2017 to September 2017. Ethical approval was taken from the Institutional Review Committee of the Institute of Medicine with reference number 326 (6-11-E).

The sample size was calculated using the following formula:

$$\begin{array}{ll} \text{n} & \text{= }{\text{Z}}^{\text{2}}\times \text{p}\times \text{(1}-\text{p)}/{\text{e}}^{\text{2}}\\ & \text{= }{\left(1.96\right)}^{\text{2}}\times 0.65\times 0.35/{(\text{0.15)}}^{\text{2}}\\ & \text{= }92 \end{array}$$

Where,

n = required sample size,
Z = 1.96 for Confidence Interval at 95%,
p = Prevalence, 65% (educated guess)
e = margin of error as 15%

Hence, we took a total of 95 patients using a convenient method of non-probability sampling.

The patients who were referred to the Department of Radiology and Imaging of TUTH for obtaining CT cerebral angiography were the study population. The CTA was performed using Siemens Somatom Definition AS+ 128 slice Multidetector Computed Tomography (MDCT) scanner of the Department of Radiology and Imaging. Study participants included all patients undergoing CT cerebral angiography in study period in TUTH for various indications. Patients who were not willing to participate in study, pregnant ladies and employees of TUTH were excluded in the study.

Data was collected in pre designed proforma in Microsoft Excel spreadsheet.

Informed written consent was taken from patients meeting the inclusion criteria.

All patients were scanned in a 128 slice MDCT scanner using standard imaging parameters of the department.

The CTA performed was evaluated. Multiplanar reconstruction images as well as 3D reformatted images in maximum intensity projections and volume rendering techniques were used for evaluation.

Completeness of arterial CoW, any variations in its arteries and any vascular pathologies seen in the images were recorded and evaluated.

Data were analysed using SPSS.

## RESULTS

Among 95 subjects included in the study, the anatomical variations in the Circle of Willis in patients undergoing CT cerebral angiography in a tertiary hospital was seen to be 52 (54.7%) while 43 (45.3%) did not show any variations. Out of the 52 cases which showed variations in CoW, variation was seen in single artery in 24 cases while the remaining 28 cases showed variations in multiple arteries.

Most common variation was hypoplastic posterior communicating artery (PCom) accounting for approximately one third of the population group 33 (34.7%) followed by fetal origin of posterior cerebral artery (PCA) 8 (8.4%). Least common variations seen were bihemispheric A2 segment of anterior cerebral artery (ACA), azygous ACA and hypoplastic anterior communicating artery (ACom) seen in one case each 1 (1.05%) (Table 1).

**Table 1:** Variations in Circle of Willis

Type of Variation	n (%)
Hypoplasia of PCom	33 (34.7)
Aplasia of PCom	7 (7.4)
Fetal origin of PCA	8 (8.4)
Hypoplastic A1 segment of ACA	4 (4.2)
Bihemispheric A2 segment of ACA	1 (1.05)
Azygous ACA	1 (1.05)
Hypoplastic ACom	1 (1.05)

Mean age of the sample population was 50 years (range: 7 to 85 years). Age groups of 50 to 60 years accounted for maximum number of cases i.e 24 (25.2%). Age groups less than 10 years accounted for the least number of cases. There was a slightly higher number of males in study 50 (52.6%) compared to females 45 (47.4%).

Most common indication for CTA in the study was for the evaluation of SAH 23 (24.2%) followed by persistent severe headache 21 (22.1%). Other indications included cases of intraparenchymal bleed 14 (14.7%), ischemic stroke 17 (17.9%), internal carotid artery (ICA) thrombosis 3 (3%), dizziness 7 (7%), post-operative cases 7 (7%) and extracranial arteriovenous malformation (AVM) 3 (3%).

Aneurysm was seen in 22 cases (Table 2). Apart from one case, all the aneurysms were seen in a single artery. One case showed two aneurysms, one in ACom and one in left ACA bifurcation.

**Table 2:** Sites of aneurysm

SiSite of aneurysm	n (%)
ACom	5 (22.73)
PCom	2 (9.09)
MCA bifurcation	5 (22.73)
Supraclinoid ICA	7 (31.82)
ACA bifurcation	1 (4.55)
P3 segment of PCA	1 (4.55)
PICA	1 (4.55)
Total	22 (100)

Besides aneurysm, 6 cases of AVM (3 intracranial and 3 extracranial) as well as 6 cases of occlusion of ICA were seen.

## DISCUSSION

Variation in arteries of CoW is common. Recognition of variation is important for neurosurgeons in surgical planning of patients with cerebral vascular pathologies including aneurysms. Though DSA is the goal standard in evaluation of patients with vascular pathologies and for demonstration of cerebral vascular variations, CTA is commonly performed due to its less invasiveness and its ability to provide 3D representation of cerebral vasculature as well as vascular pathologies with high accuracy.

In our study, 54.7% of cases showed the presence of variation in arterial circle of Willis which is comparable to cadaveric study done by S Iqbal in which 52% of cases showed variations.^1^ However, in a DSA based study done in 124 cases by Joshi et al, 40% of the cases showed the presence of variations which is slightly lower compared to results of our study.^5^

In a study done by Hamidi et al in which 500 patients who underwent MDCT, hypoplastic PCom was the most common variation seen in 34.8% of cases which was similar to our study in which hypoplasia of PCom was also the most common variation seen in 34.7% of cases. However, there were more bilateral PCom (20% vs 14.2%) and fewer unilateral PCom cases (14.7% vs 20.6%) in our study than in study by Hamidi et al.^6^ In a study done by Eftekhar et al on 102 cadavers, hypoplasia of PCom was seen in 60% of cases, 33% bilaterally and 27% unilaterally, which was higher than in our study.^7^

Second most common variation seen in our study was fetal origin of PCA which was similar to study by Hamidi et al. However, in our study, fetal origin of PCA was observed in 8.4% of cases (1 case bilateral) which was lower than in a study by Hamidi et al, in which it was seen in 19.4% of cases.^6^ Fetal origin of PCA was even more frequently seen (23.3%) in a study by Kovac et al in 455 patients undergoing MDCT. ^8^

Aplasia of PCom was seen in 7.4% cases in our study, 4.2% being bilateral and 3.2% unilateral. It was similar to findings in study by Eftekhar et al and S Iqbal. Aplastic Pcom was seen in 10% of cases in a study by Eftekhar et al (7% unilateral and 3% bilateral). It was seen in 6% of cases in a cadaveric study done by S Iqbal^1,7^

Hypoplastic A1 segment of ACA was seen in 4.2% of cases in our study which was less than the results in study by Hamidi et al (16.4%) and Kovac et al (17.6%). However, it was higher than the results seen in study by Eftekhar et al (1% cases).^6–8^

Least common variations seen in our study were bihemispheric A2 segment of ACA, Azygous ACA and Hypoplastic ACom which were seen in 1 case each in our study (1.05% each) which also showed lower incidence in studies by Hamidi et al. and Kovac et al. Bihemispheric and azygous ACA were seen in equal frequency (1.8% each) in study by Hamidi et al. Bihemispheric and azygous ACA were seen in 0.9% and 1.5% of total cases respectively in study by Kovac et al. Presence of hypoplastic ACom was however not reported in these studies.

Aplasia of A1 seen in 5.2% of cases in study by Hamidi et al. and 0.4% of cases in study by Kovac et al, was however, not seen in our study which was also not reported in study by Eftekhar et al.^6–8^

The findings of our study cannot be generalized to the whole population of Nepal as this study was conducted in a single hospital.

## CONCLUSIONS

MDCT cerebral angiography is commonly performed imaging modality for evaluation of patients with suspected vascular pathology in the brain. Anatomical variation of CoW is commonly encountered finding in patients being evaluated for cerebral vascular pathology. Multidetector computed tomography (MDCT) can often detect variations in arteries of CoW, recognition of which is crucial in operative management of vascular pathologies. Further studies with multicenter involvement is recommended to come to a more robust conclusion.
